# Assessment of the Parameters of Adaptive Cell-Mediated Immunity in Naïve Common Marmosets (Callithrix jacchus)

**Published:** 2018

**Authors:** I. V. Gordeychuk, A. I. Tukhvatulin, S. P. Petkov, M. A. Abakumov, S. A. Gulyaev, N. M. Tukhvatulina, T. V. Gulyaeva, M. I. Mikhaylov, D. Y. Logunov, M. G. Isaguliants

**Affiliations:** Chumakov Federal Scientific Center for Research and Development of Immune-and-Biological Products of Russian Academy of Sciences, premises 8, bldg. 1, Village of Institute of Poliomyelitis, Settlement “Moskovskiy”, Moscow, 108819, Russia; N.F. Gamaleya National Research Center for Epidemiology and Microbiology, Gamaleya Str., 18, Moscow, 123098, Russia; Sechenov First Moscow State Medical University, Bolshaya Pirogovskaya Str., 19, bldg. 1, Moscow, 119146, Russia; MTC, Karolinska Institutet, 171 77, Stockholm, Sweden; Pirogov Russian National Research Medical University, Ostovitjanova Str. 1, Moscow, 117997, Russia; National University of Science and Technology MISiS, Leninsky Ave., 4, Moscow, 119049, Russia; Russian Medical Academy of Continuous Professional Education, Barrikadnaja Str., 2/1, bldg. 1, Moscow, 125993, Russia; Mechnikov Research Institute for Vaccines and Sera, Maliy Kazenniy Lane, 5a, Moscow, 105064, Russia; Rīga Stradiņš University, LV-1007, Riga, Lativa

**Keywords:** adaptive cell-mediated immunity, common marmoset, flow cytometry, Callithrix jacchus

## Abstract

Common marmosets are small New World primates that have been increasingly used in biomedical research. This report presents efficient protocols for assessment of the parameters of adaptive cell-mediated immunity in common marmosets, including the major subpopulations of lymphocytes and main markers of T- and B-cell maturation and activation using flow cytometry with a multicolor panel of fluorescently labelled antibodies. Blood samples from eight common marmosets were stained with fluorescently labeled monoclonal antibodies against their population markers (CD45, CD3, CD20, CD4, CD8) and lymphocyte maturation and activation markers (CD69, CD62L, CD45RO, CD107a and CD27) and analyzed by flow cytometry. Within the CD45^+^ population, 22.7±5.5% cells were CD3– CD20^+^ and 67.6±6.3% were CD3^+^CD20–. The CD3^+^ subpopulation included 55.7±5.5% CD3^+^CD4^+^CD8– and 34.3±3.7% CD3^+^CD4–CD8^+^ cells. Activation and maturation markers were expressed in the following lymphocyte proportions: CD62L on 54.0±10.7% of CD3^+^CD4^+^ cells and 74.4±12.1% of CD3^+^CD8^+^ cells; CD69 on 2.7±1.2% of CD3^+^CD4^+^ cells and 1.2±0.5% of CD3^+^CD8^+^ cells; CD45RO on 1.6±0.6% of CD3^+^CD4^+^ cells and 1.8±0.7% of CD3^+^CD8^+^ cells; CD107a on 0.7±0.5% of CD3^+^CD4^+^ cells and 0.5±0.3% of CD3^+^CD8^+^ cells; CD27 on 94.6±2.1% of CD3^+^ cells and 8.9±3.9% CD20^+^ cells. Female and male subjects differed in the percentage of CD3^+^CD4^+^CD45RO^+^ cells (1.9±0.5 in females vs 1.1±0.2 in males; p < 0.05). The percentage of CD20^+^CD27^+^ cells was found to highly correlate with animals’ age (r = 0.923, p < 0.005). The basal parameters of adaptive cell-mediated immunity in naïve healthy marmosets without markers of systemic immune activation were obtained. These parameters and the described procedures are crucial in documenting the changes induced in common marmosets by prophylactic and therapeutic immune interventions.

## INTRODUCTION

Common marmosets (CMs; *Callithrix jacchus*) are small New World primates that have been increasingly used in the modeling of human morbidities, including infectious diseases, neuropathological disorders, and cancer [1, 2]. With regard to the susceptibility of this species to infectious diseases, it represents an exquisite non-human primate model for viral, protozoan and bacterial agents, as well as prions [3], and, hence, an ideal platform for preclinical studies of the safety and effectiveness of novel immunotherapies and vaccines [4]. Substantial advantages of using CMs in biomedical research are their small size, evolutionary closeness to humans, relative ease of maintenance, and compressed lifespan, due to which the number of animals can be scaled up quickly when the need arises and then naturally reduced when the animals are not needed [3].

The evolutionary closeness to humans makes it possible to apply the well-established research methods commonly used in human studies to CMs. However, these primates significantly differ from other nonhuman primate species in many biological aspects [5]. Immunologically, marmosets (and other Callitrichids) are exceptions to the generalized stability in MHC Class I loci [6,7]. Each Callitrichid genus exhibits its own unique set of MHC Class I genes and expresses no loci comparable to the classical MHC Class I HLA-A, -B, and -C. MHC Class I loci also appear to have limited variability and a relatively accelerated turnover between generations, resulting in a low/no inter-individual variation in the immune responses to pathogens or tumor antigens [5]. The polymorphisms in their MHC class II loci are also quite limited [8]. This makes CMs particularly sensitive to viral infections [9-11], especially to infections with oncogenic viruses, which frequently result in induction of spontaneous tumors [12-15]. Early observations of this sensitivity were confirmed by experimental infection of CMs with sarcoma viruses and lymphotropic herpes viruses [16-18]. Such spontaneously and experimentally induced tumors are directly relevant to Burkitt’s lymphoma and nasopharyngeal carcinoma in humans, making CMs a powerful model with which to test the corresponding antiviral treatments and immune interventions, including prophylactic and therapeutic vaccines against these oncogenic human viruses. Despite the outbred study groups, such studies are destined to generate coherent harmonious results due to the low variations in the immune response of individual animals.

Characterization of the effects of immune interventions, vaccine-induced responses, as well as the safety aspects of the aforementioned tests, requires a careful description of the immune status of the experimental animals in the naïve state and post-activation. One of the main methods to achieve this is flow cytometric analysis using monoclonal antibodies against cell surface and intracellular antigens. While many commercially available monoclonal antibodies used for analyzing human and non-human primate cells cross-react with the marmoset antigens, some work suboptimally and some do not to work at all [19-21].

This report presents an efficient protocol to characterize the immune status of common marmosets using flow cytometry with a multicolor panel of fluorescently labelled antibodies and its application for assessing the immune status parameters and markers of immune activation in these non-human primates.

## MATERIALS AND METHODS

Animal care and housing conditions complied with the regulations of the European Parliament and the European Council Directive on the protection of animals used for scientific purposes (2010/63/EU) and also with the National Institutes of Health Guide for Care and Use of Laboratory Animals. The animals were housed in pairs in wire mesh cages (cage size 80×55×130 cm) with wooden sleeping boxes and branches for climbing. Urine and feces were removed daily by changing the trays. Room temperature was maintained at 27±2°C, and the relative humidity was kept between 60 and 80%. Light cycle was set to a 12-hour day/night switch. The HEPA-filtered air exchange rate was set to 8 times per hour. CMs received water ad libitum and custom marmoset feed that was unchanged during the experiment. Water and food quality were controlled on a regular basis

The study protocol was approved by the Ethical Committee of the Chumakov Federal Scientific Center for Research and Development of Immune-and-Biological Products of the Russian Academy of Sciences (Chumakov FSC R&D IBP RAS, Moscow, Russia). The study included 8 animals, 3 males and 5 females, aged 23 to 48 months and weighing 360–400 grams, bred and maintained in the Experimental Clinic of Callitrichidae at the Chumakov FSC R&D IBP, RAS. All the experiments were performed by personnel certified for working with non-human primates by the Karolinska Institute (Stockholm, Sweden). The conditions of housing and maintenance of the animals remained unchanged throughout the experiment. No adverse events were detected in the subject animals during the experiment and in the two-week follow-up period after the procedure. All animals were identified using subcutaneous radio-frequency chips with unique 15-digit codes (Globalvet, Moscow, Russia). The IDs in tables and figures represent the last four digits of the code.

Venous blood samples (2 ml) were obtained from eight CMs by femoral vein puncture using a 2.5 ml syringe with a 25G needle pre-filled with 25 IU of sodium-heparin (Belmedpreparaty, Minsk, Belarus) per ml. Aliquots of 50 µl of whole blood per test were incubated for 30 min at 22o C with pre-titrated amounts of the following antibodies: PE mouse anti-marmoset CD45 (BioLegend, San Diego, USA, clone 6C9, cat. 250204); Alexa Fluor 700 mouse anti-human CD3 (BD, New Jersey, USA, clone SP34-2, cat. 557917); FITC mouse anti-human CD20 (Beckman Coulter, Brea, USA, clone H299, cat. 6602381); PerCP-Cy5.5 mouse anti-human CD4 (BD, clone L200, cat. 552838); PE anti-marmoset CD8 (BioLegend, clone 6F10, 250304); APC mouse anti-human CD69 (BD, clone L78, cat. 654663); BV421 mouse anti-human CD62L (BD, clone SK11, cat. 743207); PE/Cy7 anti-human CD45RO (BioLegend, clone UCHL1, cat. 304230); BV421 mouse anti-human CD107a (BD, clone H4A3, cat. 562623); and APC anti-human CD27 (BioLegend, clone M-T271, cat. 356409). After incubation with the given antibodies, samples were treated with 1 ml of RBC lysis buffer (BioLegend, cat. 420301) for 15 min at RT and washed once with 1 ml PBS at 2000G. Samples were analyzed on a BD FACS Aria III flow-cytometer (BD) within 30 min after staining. The reactivity of each monoclonal antibody was defined as the percentage of positively stained cells relative to cells stained with all other antibodies except for the one tested (FMO control).

Statistical analysis of the data was performed using the t-test for normally distributed values, and nonparametrical Mann-Whitney and Spearman raking tests, all performed with the help of STATISTICA AXA 10 (TIBCO Software, USA).

## RESULTS

**Fig. 1:**
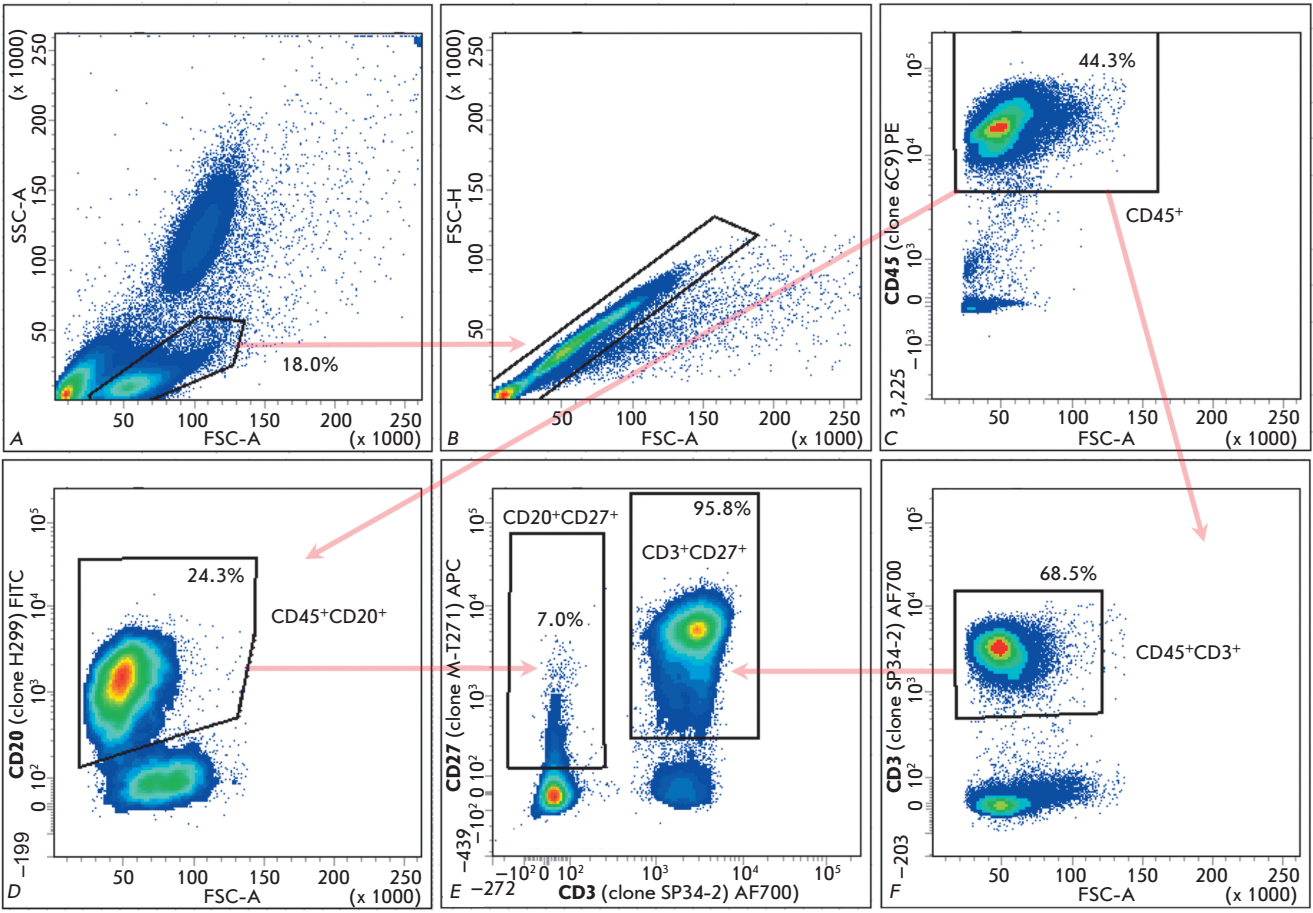
Gating and cell staining patterns for the T- and B-cell populations of a naïve CM (ID 4540). FSC-A/SSC-A population separation plot (a) and exclusion of non-single cells (b) were used for CM CD45^+^ leukocytes gating (c). The proportions of stained CD45^+^CD20^+^ (d) and CD45^+^CD3^+^ (f) cells are shown in respective gates as fractions of CD45^+^. The proportions of stained CD3^+^CD27^+^ and CD20^+^CD27^+^ cells (e) are shown in respective gates as fractions of CD3^+^ cells and CD20^+^ cells. The reactivity of each monoclonal antibody was defined as the percentage of positively stained cells relative to cells stained with all other antibodies except for the one tested (FMO control). A total of 150,000 events were processed in each measurement

**Fig. 2:**
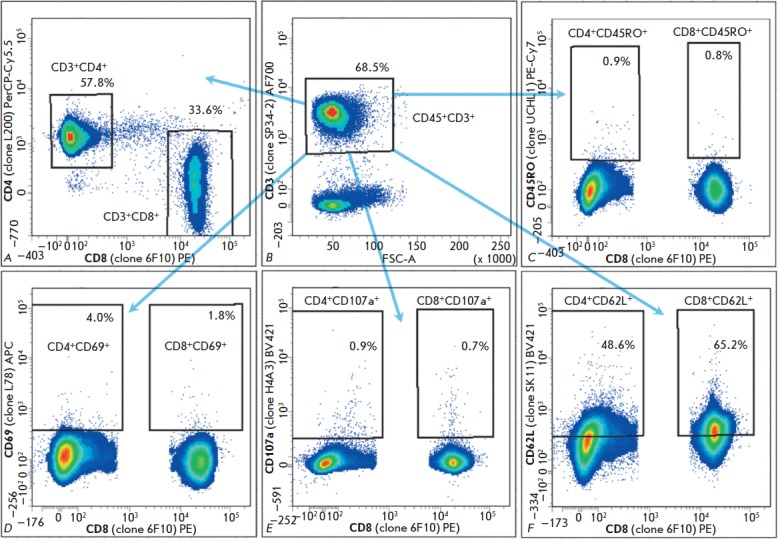
Staining patterns of cell maturation and activation markers of a naïve CM (ID 4540). The proportions of stained CD3^+^CD4^+^ and CD3^+^CD8^+^ (a) cells are shown in respective gates as fractions of CD45^+^CD3^+^ (b) cells. The proportions of stained CD45RO^+^ (c), CD69^+^ (d), CD107a^+^ (e), and CD62L^+^ (f) cells are shown in respective gates as fractions of CD3^+^CD4^+^ and CD3^+^CD8^+^ cell populations. The reactivity of each monoclonal antibody was defined as a percentage of positively stained cells relative to the cell populations stained with all other antibodies except for the one tested (FMO control). A total of 150,000 events were processed in each measurement

Few studies published so far have addressed the applicability of different commercially available monoclonal antibodies to the flow cytometry (FACS) of CM cells [19-21]. Here, we have elaborated an efficient protocol for characterizing the immune status of CMs using FACS with a multicolor panel of fluorescently labelled antibodies specific to the major subpopulations of lymphocytes and markers of T- and B-cell maturation and activation. Using this method, we characterize the immune status of naïve CMs with respect to the percentage of basic T- and B-lymphocyte subpopulations (CD45^+^, CD45^+^CD3–CD20^+^, CD45^+^CD3^+^CD20–, CD3+CD4^+^CD8–, CD3^+^CD4–CD8^+^) and the level of expression of the maturation and activation markers (CD27, CD62L, CD69, CD45RO, CD107a) on these T-and B-lymphocytes. The gating strategy and staining patterns are shown in *Fig. 1* and *Fig. 2* on the example of one naïve CM (ID 4540).

The proportions of peripheral blood cells of individual CMs labelled with receptor-specific antibodies are summarized in Table 1. CD45^+^ leukocytes accounted for 54.3±11.8% of total cells after RBC lysis. Within the CD45+ population, 22.7±5.5% were B-cells (CD45^+^CD3^–^CD20^+^) and 67.6±6.3% were T-cells (CD45^+^CD3^+^CD20^–^). The CD3^+^ subpopulation was comprised of 55.7±5.5% T-helper cells (CD3+CD4+CD8–) and 34.3±3.7% of cytotoxic T-cells (CD3^+^CD4^–^CD8^+^). The proportions of B- and T-cells, including the CD4^+^ and CD8^+^ populations, found in this study corroborate earlier findings for naïve marmosets [20,22], as well as the reported values for a healthy human population [23, 24].

**Table 1:** Proportions of reactive peripheral blood cells of naïve CMs

Parameter	Marmoset ID, parameter %										Total, M±σ, %
	Female						Male				
	2996	2998	0519	3016	2997	M±σ	2994	4540	4520	M±σ	
Age, months	29	29	23	48	25	30.8±10.0	30.0	25.0	25.0	26.7±2.9	29.3±8.0
*CD45^+^	67.5	64.5	62.3	43.5	43.2	56.2±11.9	42.1	44.3	66.6	51.0±13.6	54.3±11.8
CD45^+^CD3^-^CD20^+^	28.7	32.4	17.7	17.5	20.4	23.3±6.8	22.3	24.3	18.4	21.7±3.0	22.7±5.5
CD45^+^CD20^+^CD27^+^	8.3	11.8	5.9	17	7.9	10.2±4.4	8.9	7.0	4.7	6.9±2.1	8.9±3.9
CD45^+^CD3^+^CD20^-^	62.4	57.6	69.6	74.7	64.4	65.7±6.6	66.5	68.5	76.9	70.6±5.5	67.6±6.3
CD45^+^CD3^+^CD27^+^	93.9	93.2	96.2	98.4	93.2	95.0±2.3	91.8	95.8	94.6	94.1±2.1	94.6±2.1
CD3^+^CD4^-^CD8^+^	39.2	32.7	34.4	40	32.9	35.8±3.5	33.2	33.6	28.5	31.8±2.8	34.3±3.7
CD3^+^CD8^+^CD62L^+^	72.7	81.2	89.3	86.7	51.8	76.3±15.1	76.4	65.2	72.0	71.2±5.6	74.4±12.1
CD3^+^CD8^+^CD69^+^	0.9	1.1	1.6	1.9	0.3	1.2±0.6	1.2	1.8	1.0	1.3±0.4	1.2±0.5
CD3^+^CD8^+^CD45RO^+^	2	2.4	1.8	1.8	0.8	1.8±0.6	2.0	0.8	0.7	1.2±0.7	1.8±0.7
CD3^+^CD8^+^CD107a^+^	0.9	0.5	0.8	0.5	0	0.5±0.4	0.2	0.7	0.2	0.4±0.3	0.5±0.3
CD3^+^CD4^+^CD8^-^	49.9	57.7	51.2	49.7	57.8	53.3±4.1	55.5	57.8	66.1	59.8±5.6	55.7±5.5
CD3^+^CD4^+^CD62L^+^	47.3	56	73.8	66	43	57.2±12.8	49.1	48.6	47.8	48.5±0.7	54.0±10.7
CD3^+^CD4^+^CD69^+^	1.1	2.3	3.8	4.2	1.7	2.6±1.3	2.0	4.0	2.7	2.9±1.0	2.7±1.2
CD3^+^CD4^+^CD45RO^+^	2	1.7	2.3	2.4	1.1	1.9±0.5**	1.3	0.9	1.0	1.1±0.2**	1.6±0.6
CD3^+^CD4^+^CD107a^+^	1.2	0.6	1.5	0.9	0.2	0.9±0.5	0.2	0.9	0.4	0.5±0.4	0.7±0.5

^*^ – within lymphocyte population gated on a FSC-A/SSC-A plot and non-single cells excluded

^**^ – values with statistically significant differences (p < 0.05)

Lymphocyte activation and maturation markers were expressed in the immune cell subpopulations specified above in the following proportions: CD62L (L-selectin; lymphoid system homing signal, cleaved following cell activation) on 54.0±10.7% of CD3^+^CD4^+^ cells and 74.4±12.1% of CD3^+^CD8^+^ cells; CD69 (early T-cell activation marker) on 2.7±1.2% of CD3^+^CD4^+^ cells and 1.2±0.5% of CD3^+^CD8^+^ cells; CD45RO (memory-activated T-cells) on 1.6±0.6% of CD3^+^CD4^+^ cells and 1.8±0.7% of CD3^+^CD8^+^ cells; CD107a (T-cell activation) on 0.7±0.5% of CD3^+^CD4^+^ cells and 0.5±0.3% of CD3^+^CD8^+^ cells; CD27 (TNF receptor superfamily member (TNFRSF7); and memory B-cells, mature T-cells) on 94.6±2.1% of T-cells (CD20^–^CD3^+^). The values lay in the range of the ones observed in the recently published unique study of the distribution of diverse immune cell populations/subpopulations by Neumann *et al*. [21].

Interestingly, however, we observed a lower, compared to the published data [21], proportion of CD45^+^CD20^+^CD27^+^ memory B-cells (8.9±3.9%), indicating a low level of B-cell activation. We explained this by the fact that the mean age of the animals used in our study was lower compared to the study by Neumann *et al*. (29.3±8.0 months) (*Table 1*). Besides, the proportion of subpopulations of CD62L^+^CD4^+^ and CD62L^+^CD8^+^ T-cells determined in this report appeared to be lower than the respective values described by Yoshida *et al*. [25], which might indicate T-cell activation. The percentage of CD20^+^CD27^+^ cells (activated B-cells) correlated with the percentage of CD62L-positive (non-activated) CD3^+^CD4^+^, but not CD3^+^CD8^+^ T-cells (Spearman Ranking test; r=0,902; p=0,006). No other signs of systemic immune activation were observed.

The composition of the lymphocyte subpopulations and the levels of activation markers of T- and B-cells did not differ for male and female subjects. The only statistically significant difference was found in the proportion of the reactive CD3^+^CD4^+^CD45RO^+^ cells (1.9±0.5 in females vs 1.1±0.2 in males; t-value = 2.5658, df=6, p=0,0426; t-test). The observed levels of CD3^+^CD4^+^CD45RO^+^ cells in both males and females were within the values previously reported for naïve healthy animals [20].

The animals were of different age; one CM was considerably older than the others in the group (ID 3016, Table 1). In view of this, we analyzed the age dependence of all immune parameters. The proportion of CD45^+^CD20^+^CD27^+^ memory B-cells was found to highly correlate with animals’ age (Spearman ranking test, r = 0.923, p = 0.0011). Furthermore, the correlation was still significant if this single older animal was removed from the analysis (r = 0.798; p = 0.03). This correlation supports our hypothesis that a lower percentage of B-cells in our study is observed due to the younger mean age of the animals used. An analysis of other parameters of the immune status and activation markers revealed no significant age-related differences (p > 0.05).

## DISCUSSION

In this report, we define the basal characteristics of the status of the immune system of CMs, typical of naïve healthy animals of differing age and gender, which are necessary for identifying the changes induced by the disease, as well as by immune therapy and/or vaccination. We observed that young animals had a lower proportion of CD45^+^CD20^+^CD27^+^ memory B-cells compared to the published data [21], which is indicative of the low level of B-cell activation. The relevance of these observations to other juvenile and sub-adult animals will be addressed in further studies. Aside from this, we observed no statistically significant age-related changes neither in the parameters of immune status nor in the markers of immune differentiation, which allowed us to assume that CMs older than two years are suitable for immune testing in mixed-age groups.

The proportion of subpopulations of CD62L positive CD4^+^ and CD8^+^ T-cells determined in this report was lower than the respective values described by Yoshida *et al*. [25]. L-selectin (CD62L) mediates T-cell entry into the lymph nodes. The L-selectin levels are down-regulated in T-cells transmigrating within the lymph nodes, while its levels on the T cells in non-lymphoid organs and blood remain unchanged [26]. During T-cell activation, L-selectin expression reduces to 10% of the initial level within several minutes by ectodomain shedding [27]. The decrease in the proportion of CD62L^+^ T-cells indicates, therefore, a possible recent/on-going T-cell activation. Interestingly, expression of CD27^+^ on the B-cells of CMs correlated with the expression of L-selectin/CD62L^+^ by CD4^+^ T-cells (p < 0.01): i.e., B-cell activation was associated with the absence of immune activation (no CD62L shedding) in CD4^+^ T cells. Earlier reports described associations between the expression of surface activation markers of memory B-cells CD27 and CD21 [28]. Complement receptor type II CD21 is expressed on most of the mature B-cells; earlier papers demonstrated that shedding of CD21 by B-cells occurs simultaneously with shedding of CD62L by the naïve and memory lymphocytes, the latter required to recruit them to the sites of the infection [29]. Both processes appear to be driven by the same family of proteases [29]. These data help to define the mechanism of CD21-mediated correlation between the expression of the B-cell CD27 activation marker and CD62L on T-cells. The correlation between the expression of CD27 by B-cells designating their activation, and of CD62L by CD4^+^ T-cells (actually, an inverse correlation with CD62L shedding, designating CD4^+^ T cell activation), may reflect the concordant regulation of the differentiation of these immune cell subsets in non-human primates.

In conclusion, we have characterized basal parameters of the immune status of naïve healthy marmosets without markers of systemic immune activation. Knowledge of these parameters is crucial for documenting the changes induced in CMs by therapeutic and prophylactic interventions. The antibody panel and gating procedures elaborated here allowed for a reliable quantification of specific immune cell populations and assessment of their functional status. Therefore, they could be recommended for use in trials of novel immune interventions, such as vaccines against chronic viral infections and cancer, in CMs.

## Abbreviations

CD: cluster of differentiation
FMO: fluorescence minus one control
FSC: forward-scattered light
HEPA: high-efficiency particulate air
HLA: human leukocyte antigen
M±?: mean value ± standard deviation
IU: International Unit
MHC: major histocompatibility complex
SSC: side-scattered light
